# PGAGP: Predicting pathogenic genes based on adaptive network embedding algorithm

**DOI:** 10.3389/fgene.2022.1087784

**Published:** 2023-01-20

**Authors:** Yan Zhang, Ju Xiang, Liang Tang, Jialiang Yang, Jianming Li

**Affiliations:** ^1^ School of Computer Science and Engineering, Central South University, Changsha, China; ^2^ School of Information Science and Engineering, Changsha Medical University, Changsha, China; ^3^ Academician Workstation, Changsha Medical University, Changsha, China; ^4^ School of Computer and Communication Engineering, Changsha University of Science and Technology, Changsha, China; ^5^ Department of Basic Medical Sciences and Neuroscience Research Center, Changsha Medical University, Changsha, China; ^6^ Qingdao Geneis Institute of Big Data Mining and Precision Medicine, Qingdao, China; ^7^ Geneis Beijing Co., Ltd, Beijing, China

**Keywords:** disease-gene prediction, biological network, network embedding, network propagation, random projection

## Abstract

The study of disease-gene associations is an important topic in the field of computational biology. The accumulation of massive amounts of biomedical data provides new possibilities for exploring potential relations between diseases and genes through computational strategy, but how to extract valuable information from the data to predict pathogenic genes accurately and rapidly is currently a challenging and meaningful task. Therefore, we present a novel computational method called PGAGP for inferring potential pathogenic genes based on an adaptive network embedding algorithm. The PGAGP algorithm is to first extract initial features of nodes from a heterogeneous network of diseases and genes efficiently and effectively by Gaussian random projection and then optimize the features of nodes by an adaptive refining process. These low-dimensional features are used to improve the disease-gene heterogenous network, and we apply network propagation to the improved heterogenous network to predict pathogenic genes more effectively. By a series of experiments, we study the effect of PGAGP’s parameters and integrated strategies on predictive performance and confirm that PGAGP is better than the state-of-the-art algorithms. Case studies show that many of the predicted candidate genes for specific diseases have been implied to be related to these diseases by literature verification and enrichment analysis, which further verifies the effectiveness of PGAGP. Overall, this work provides a useful solution for mining disease-gene heterogeneous network to predict pathogenic genes more effectively.

## 1 Introduction

Diseases have been threatening human health and life for a long time. As we know, many complex diseases are closely related to the mutations and dysfunction of pathogenic genes. Accurate identification of pathogenic genes is very important for the mechanism research of complex diseases and their diagnosis and treatment (do Valle Í, 2020; Menche et al., 2015). Traditional methods, e.g., linkage mapping and genome-wide association, are helpful for finding disease genes, but their candidate lists still contain hundreds or thousands of genes, needing expensive experiments to further determine disease genes (Hindorff et al., 2009; Johnson and O'Donnell, 2009; Ott et al., 2015; Shim et al., 2017). So, in the last decades, a large number of computational methods have been introduced to infer pathogenic genes (Zeeshan et al., 2020; Ata et al., 2021; Xiang et al., 2021a; Ruan and Wang, 2021; Xiang et al., 2022a).

Thanks to a variety of high-throughput experimental techniques, protein-protein interactions as well as pathogenic association data are growing rapidly (Liu et al., 2020; Xiang et al., 2022b). Therefore, network-based methods are one of the most popular methods for disease-gene prediction (Köhler et al., 2008; Li and Patra, 2010; Vanunu et al., 2010; Xie et al., 2012; Luo et al., 2021). The protein-protein interactions (PPI) are a popular biological data resource widely used in disease-gene prediction and related issues (Oti et al., 2006; Köhler et al., 2008; Li and Patra, 2010; Hu et al., 2018; Meng et al., 2022). For example, the random walk with restart (RWR) on a PPI network was proposed to predict disease genes (Köhler et al., 2008), which uses a random walk process to explore the network proximity between candidate genes and seed genes (i.e., known pathogenic genes of a disease). However, any existing single-source data, due to data noise and other problems, is difficult to fully reflect the relevant information between diseases and genes. Many relevant studies have revealed that genes associated with the same or similar diseases are generally related functionally, which are adjacent to or close to each other in the PPI network. Therefore, the comprehensive use of pathogenic and gene-related information can improve the prediction performance. For instance, the RWR algorithm was extended into a disease-gene heterogeneous network, resulting in the popular RWRH algorithm (Li and Patra, 2010). Based on the similar heterogeneous network, Vanunu et al. (2010) proposed the algorithm called PRINCE, and Xie et al. (2012) presented the BiRW algorithm to globally prioritize pathogenic genes for all diseases simultaneously.

Many disease-gene-prediction methods with the heterogeneous network have been presented, but how to extract valuable information from the network to predict pathogenic genes accurately and rapidly is currently a challenging and meaningful task. Some researchers recently have carried out relevant work by integrating novel network embedding techniques (Han et al., 2018; Zhou et al., 2020; Xiang et al., 2021b). For example, the PrGeFNE method was proposed for predicting disease-related genes by using fast network embedding (Xiang et al., 2021b). Network embedding (NE) or network representation learning (NRL) has become an effective strategy to mine useful information from the network data (Han et al., 2018). At present, a variety of network embedding algorithms have been presented, and the existing learning-based algorithms can achieve good results in many tasks such as node classification and link prediction (Wang et al., 2016; Cunchao et al., 2017; Zhang et al., 2018; He et al., 2021; HU et al., 2021; Pio-Lopez et al., 2021). However, with the increase of network scale, the existing network embedding methods have computing bottlenecks. In order to address this problem, Gaussian random projection as a new and effective technique was applied to learn low-dimensional features of nodes from a large-size network, but some key information of network structure may be lost, due to the limit of dimension, resulting in the degradation of algorithm performance (Zhang et al., 2018; HU et al., 2021).

Therefore, we present a type of novel algorithms for inferring pathogenic genes based on adaptive random projection (PGAGP). First, we propose an adaptive algorithm based on Gaussian random projection (AGP) for extracting the features of nodes from a large-scale heterogeneous network. It first generates the raw features of nodes from the heterogeneous network by Gaussian random projection and then will optimize these raw features by an adaptive refining process to generate the final low-dimensional feature matrix of nodes. Then, we use the extracted feature matrix to improve the disease-gene heterogenous network, and we apply network propagation process to the improved heterogenous network to mine potential pathogenic genes more effectively.

In the following, Section 2 will introduce the used datasets and the details of the PGAGP method, including the AGP algorithm, the method of improving disease-gene heterogenous network, the strategies of integrating adaptive random projection. In Section 3, we study the effect of PGAGP’s parameters and the integrated strategies on predictive performance and evaluate the performance of the PGAGP method by a serial of experiments, along with the comparison of AGP with other state-of-the-art network-embedding algorithms and the case studies for specific diseases. Finally, we present our conclusion.

## 2 Materials and methods

### 2.1 Dataset

In order to evaluate the validity of our method, we use the multiple kinds of biological network data, including a disease-gene network (DGN), a PPI network, and a disease-disease network (DDN). These biological networks are described in detail as follows. For the PPI network, the comprehensive interactome originally collected by Menche et al. will be used, which was derived from several high-quality databases (e.g., HPRD, IntAct and PINA) (Menche et al., 2015). The DGN network is obtained from DisGeNet (Piñero et al., 2017), which is a discovery platform containing a large number of human disease/phenotype-related variants and genes. We filtered the raw disease-gene association data by selecting the “disease” and ‘Disease or Syndrome’ types in DisGeNet. Then, we filtered out genes that are not in the PPI network. The DDN network is derived by using the disease-disease similarity scores calculated recently by the same method in MimMiner (van Driel et al., 2006), and we map the OMIM IDs to the UMLS IDs in DisGeNet. Finally, the GGN network contains 13,271 nodes, the DDN network contains 7,003 nodes, the DGN network has 15,786 disease-gene associations, while the resulting heterogeneous network of genes and diseases contains 20,274 nodes and 345, 962 edges.

### 2.2 Method

**FIGURE 1:**
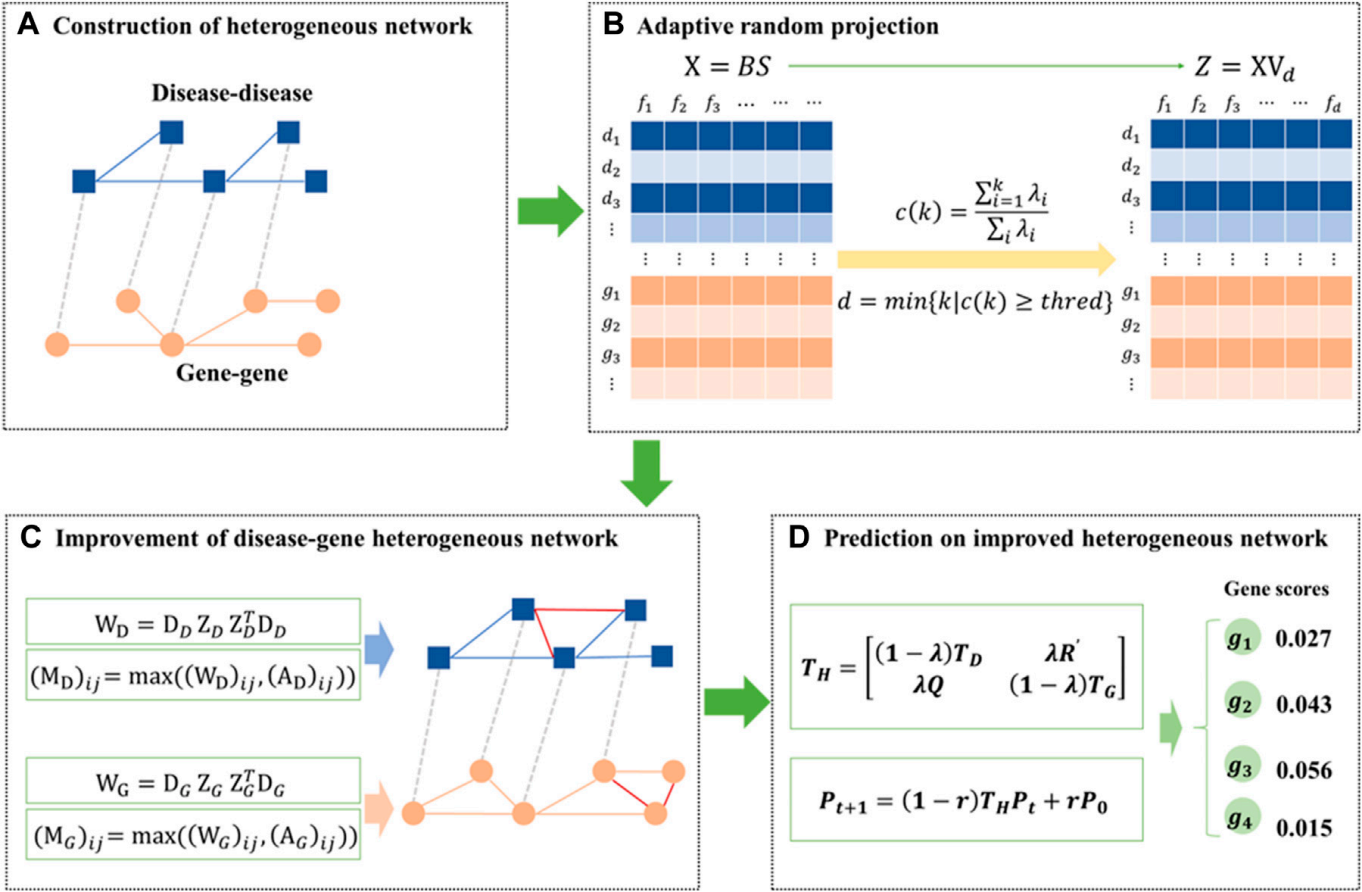
Overview of the PGAGP algorithm **(A)** A disease-gene heterogeneous network consisting of a disease-gene network, a PPI network and a disease-disease network **(B)** The heterogeneous network is fed to adaptive random projection algorithm to extract the features of nodes (diseases and genes) **(C)** The PPI network and the disease-disease network are improved by using the above extracted features, and thus an improved disease-gene heterogeneous network is constructed **(D)** The improved disease-gene heterogeneous network is fed to a network propagation algorithm to infer pathogenic genes more effectively.

In this work, a disease-gene-prediction method called PGAGP will be proposed based on adaptive Gaussian random projection. This method consists of the following steps. Starting from a disease-gene heterogeneous network (DGH) consisting of disease-related and gene-related associations, 1) we propose an adaptive Gaussian random projection algorithm, so as to obtain the features of nodes (diseases and genes) from the DGH network; 2) we improve the disease-gene heterogeneous network by using the extracted features; 3) we predict pathogenic genes on the improved DGH network (see Figure 1).

#### 2.2.1 Adaptive Gaussian random projection

In order to more effectively make use of known biological associations to infer potential disease-related genes, we will first construct a disease-gene heterogeneous network by integrating the know disease-gene associations, disease-disease associations, and gene-gene associations. Then, we extract the low-dimensional features of network nodes to mine valuable information from the disease-gene heterogeneous network. The low-dimensional features of diseases and genes can be used to directly infer disease-gene associations, e.g., by the similarity between feature vectors. Or they can also be used to improve the structure of the original disease-gene heterogeneous network, so as to improve the performance of disease-gene prediction. In this scenario, the algorithm of extracting the features of network nodes is critical to disease-gene prediction. So, we propose the following adaptive Gaussian random projection algorithm (AGP).

Generally, we use the adjacency matrix of a network $A\in {\left\{0,1\right\}}_{n\times n}$ to represent the network. If there are edges between nodes $v_{i}$ and $v_{j}$ , then $A_{ij}=1$ , otherwise $A_{ij}=0$ . To predict disease-related genes, we have constructed a DGH network that consists of a DGN network, a PPI network and a DDN network. To effectively mine the valuable information from the DGH networks, the adaptive Gaussian random projection algorithm (AGP) is proposed to obtain the features of network nodes.

For an undirected graph, an objective function can be defined by, $\min_{X}{\left\|\Phi \left(A\right)-X∙X^{T}\right\|}_{2}$ , where A is the adjacent matrix of the graph, $\Phi \left(A\right)\in R^{n\times n}$ is a targeted similarity function of A , $X\in R^{n\times d^{\prime }}$ denotes a (relatively low-dimensional) feature matrix, n denotes the number of nodes, $d^{\prime }=p*n$ denotes the dimension of initial features, and *p* denotes the proportion of initial dimension. Specifically, $\Phi \left(A\right)$ is a higher-order matrix of A, and can be formulated as $\Phi \left(A\right)=B∙B^{T}$ , where $B=\sum_{r=0}^{r=q}\alpha_{r}{\tilde{A}}^{r}$ , $\tilde{A}={\tilde{D}}^{-\frac{1}{2}}A{\tilde{D}}^{-\frac{1}{2}}$ , $\tilde{D}$ denotes a diagonal matrix with ${\tilde{D}}_{ii}=\sum_{j}A_{ij}$ , and $\alpha_{r}$ denotes the pre-defined weight of the r-th matrix.

As we know, the optimal solution of the above objective function can be obtained by singular value decomposition (SVD), but it is not suitable for large-scale networks due to its high computing consumption (Eckart and Young, 1936). Differently from SVD and other methods of direct parameter optimization, we here apply Gaussian random projection (GRP) to extract the initial feature matrix X , due to its rapidity and effectiveness. First, a subspace $S\in R^{n\times d^{\prime }}$ is generated by Gaussian distribution $S_{ij}∼N\left(0,\frac{1}{d^{\prime }}\right)$ . The subspace S contains of a group of standard basis vectors that are orthogonal to each other, i.e., $S^{T}S=I$ . Based on the standard basis vectors, then, X can be obtained by mapping B into the subspace,

$$X=B∙S=\sum_{r=0}^{q}\alpha_{r}{\tilde{A}}^{r}S=\sum_{r=0}^{q}\alpha_{r}S_{r}$$ (1)

where $S_{r}=\tilde{A}S_{r-1}$ and $S_{0}=S$ .

To further optimize the feature matrix X , a post-processing process is applied. Specifically, we first generate the column-centered matrix Y by $Y_{ij}=X_{ij}-\sum_{k}X_{kj}$ , and then obtain the eigenvalues and corresponding eigenvectors of $Y^{T}Y$ ,

$$LVS=\left\{\left(\lambda_{i},\;v_{i}\right)\;|\;i=1,2,\ldots \right\}$$ (2)

where $\lambda_{i}\geq \lambda_{i+1}$ . And then, we map the feature matrix X into the new eigen space by $Xv_{i}$ , and $\lambda_{i}$ corresponds to the contribution of the dimension i . We define the relatively accumulative contribution from $v_{1}$ to $v_{k}$ as

$$c\left(k\right)=\sum_{i=1}^{k}\lambda_{i}/\sum_{i}\lambda_{i},$$ (3)

and we define the feature dimension of final feature matrix as the minimum of k when satisfying, $c\left(k\right)\geq threshold$ , that is, $d=\min\left\{k|c\left(k\right)\geq threshold\right\}$ , where threshold denotes the threshold of relatively accumulative contribution. As a result, the final feature matrix can be obtained by $Z=XV_{d}$ , where the projection space is defined as $V_{d}=\left(v_{1},v_{2},\ldots ,v_{d}\right)$ .

#### 2.2.2 Improvement of DGH network

After extracting the final features of nodes, we improve the DDN network and the PPI network by using the extracted information. Specifically, we first extract the feature matrix $Z_{D}$ of diseases from the above final feature matrix Z, and calculate the similarity scores between diseases by,

$$W_{D}=D_{D}Z_{D}Z_{D}^{T}D_{D},$$ (4)

where $D_{D}$ denotes a diagonal matrix of diseases with ${\left(D_{D}\right)}_{ii}={\left(Z_{D}Z_{D}^{T}\right)}_{ii}^{-1/2}$ . Then, we extract the feature matrix $Z_{G}$ of genes from Z, and calculate the similarity scores between genes by,

$$W_{G}=D_{G}Z_{G}Z_{G}^{T}D_{G},$$ (5)

where $D_{G}$ denotes a diagonal matrix of genes with ${\left(D_{G}\right)}_{ii}={\left(Z_{G}Z_{G}^{T}\right)}_{ii}^{-1/2}$ . Further, we obtain sparse matrices of $W_{D}$ and $W_{G}$ , corresponding to the reconstructed disease network (DNrc) and the reconstructed gene network (GNrc).

The original DDN network and PPI network are often incomplete. The DNrc and GNrc networks contain the refined information that extracts from the original DGH network, which may be helpful for inferring potential disease genes. So, we further generate the improved disease network (DNim) by defining the new matrix $M_{D}$ of diseases,

$${\left(M_{D}\right)}_{ij}=\max\left({\left(W_{D}\right)}_{ij},{\left(A_{D}\right)}_{ij}\right)).$$ (6)

And, similarly, we generate the improved gene network (GNim) by defining new matrix $M_{G}$ of genes,

$${\left(M_{G}\right)}_{ij}=\max\left({\left(W_{G}\right)}_{ij},{\left(A_{G}\right)}_{ij}\right)).$$ (7)

The disease-gene heterogeneous network is a useful network framework for network-based disease-gene prediction, but the original heterogeneous network is not ideal due to the noise in the original DDN network and PPI network. To provide better network structure for disease-gene prediction, we consider the following two kinds of improved disease-gene heterogeneous networks (HNim and HNrc).

**HNim:** First, we propose the improved disease-gene heterogeneous network that is constructed by integrating the above DNim and GNim networks with known disease-gene association network. This will provide a better heterogeneous network framework for disease-gene prediction.

**HNrc:** As a compared strategy, we also generate a reconstructed DGH network by directly integrating the above DNrc and GNrc with known disease-gene associations.

Moreover, as comparison, we also construct an original disease-gene heterogeneous network by integrating the original DDN network and the original PPI network with known disease-gene associations (see Section 3).

#### 2.2.3 Disease-gene prediction integrating adaptive random projection

In this work, we study three kind strategies for disease-gene prediction integrating adaptive random projection. The first and second strategies will conduct network propagation on HNim and HNrc heterogeneous networks, respectively. The third strategy will infer disease-gene associations by the cosine similarity scores between feature vectors of diseases and genes.

##### 2.2.3.1 PGAGP_HNim: Prediction on HNim heterogeneous network

To make use of the above improved disease-gene heterogeneous network called HNim to infer potential disease genes, we will apply a random walk process to the improved HNim network, due to its good performance in many cases. First, we obtain the column-normalization matrices ( $T_{D}$ , $T_{G}$ , $R^{\prime }$ , and Q ) of $M_{D}$ , $M_{G}$ , R , and $R^{T}$ , where R denotes the known disease-gene association matrix and $R^{T}$ denotes the transpose matrix of R. Then, the probability transition matrix on the HNim network is denoted as,

$$T_{H}=\left[\begin{array}{cc} {\left(1-\lambda \right)T}_{D} & \lambda R^{\prime }\\ \lambda Q & \left(1-\lambda \right)T_{G} \end{array}\right],$$ (8)

where λ denotes the inter-layer jump probability (note that considering the possible existence of isolated nodes in the HNim network, we usually conduct further column-normalization on $T_{H}$ ). A random walker in a network layer (e.g., the disease network layer) may jump to another network (e.g., the gene network layer) with probability λ , or it may stay at the current layer with probability of 1−λ .

The stable solution of the random walk process on the network can be obtained by,

$$P_{t+1}=\left(1-r\right)T_{H}P_{t}+rP_{0}$$ (9)

where the initial probability vector $P_{0}={\left[D_{D}^{T},\;Q^{T}\right]}^{T}$ , and $D_{D}$ is a diagonal matrix of diseases. The difference of probability vectors at different time steps becomes negligible after a small number of iterations, and the stable probability vector $P_{\infty }={\left[D_{\infty }^{T},\;Q_{\infty }^{T}\right]}^{T}$ is reached, which denotes the closeness between candidate genes and the seed gene(s). Each column of $P_{\infty }$ records the disease-relevance scores of all genes with a given disease.

##### 2.2.3.2 PGAGP_HNrc: Prediction on HNrc heterogeneous network

As comparison, we also apply the above random-walk process to the HNrc heterogeneous network to infer candidate genes of diseases. First, the column-normalization matrices ( $W_{D}^{\prime }$ , $W_{G}^{\prime }$ , $R^{\prime }$ and Q ) of $W_{D}$ , $W_{G}$ , R , and $R^{T}$ , where R also denotes the known disease-gene association matrix. Then, the probability transition matrix on the HNrc network can be obtained by

$$T_{H}=\left[\begin{array}{cc} {\left(1-\lambda \right)W^{\prime }}_{D} & \lambda R^{\prime }\\ \lambda Q & \left(1-\lambda \right)W_{G}^{\prime } \end{array}\right].$$ (10)

Then, as in PGAGP_HNim, we conduct the network propagation process based on this probability transition matrix to generate the disease-relevance scores of all genes.

##### 2.2.3.3 PGAGP_CSim: Prediction based on cosine similarity of features

The extracted features of nodes contain useful information that reflect the characteristics of genes and diseases. The similarity of characteristics between genes and diseases can reflect the relatedness between them. Therefore, we also use the cosine similarity between the node features to evaluate the disease-relevance scores between genes and specific diseases. Specifically, for a disease vector and a gene vector, the disease-relevance score between them is calculated by,

$$CSim\left(d_{i},\;g_{j}\right)=\frac{Z_{D}\left(d_{i},•\right)Z_{G}^{T}\left(g_{j},•\right)}{\left|Z_{D}\left(d_{i},•\right)\right|∙\left|Z_{G}\left(g_{j},•\right)\right|},$$ (11)

where $Z_{D}\left(d_{i},•\right)$ is a row vector in the feature matrix $Z_{D}$ of diseases, $Z_{G}\left(g_{j},•\right)$ is a row vector in the feature matrix $Z_{G}$ of genes. For a given disease, we calculate disease-relevance scores of all candidate genes, and then we obtain a list of candidates for this disease by the decreasing order of disease-relevance scores. Algorithm 1 describes the PGAGP method for predicting potential disease-gene associations.

Algorithm 1 PGAGP ( $A_{D}$ , $A_{G}$ , R, p, threshold, STR)

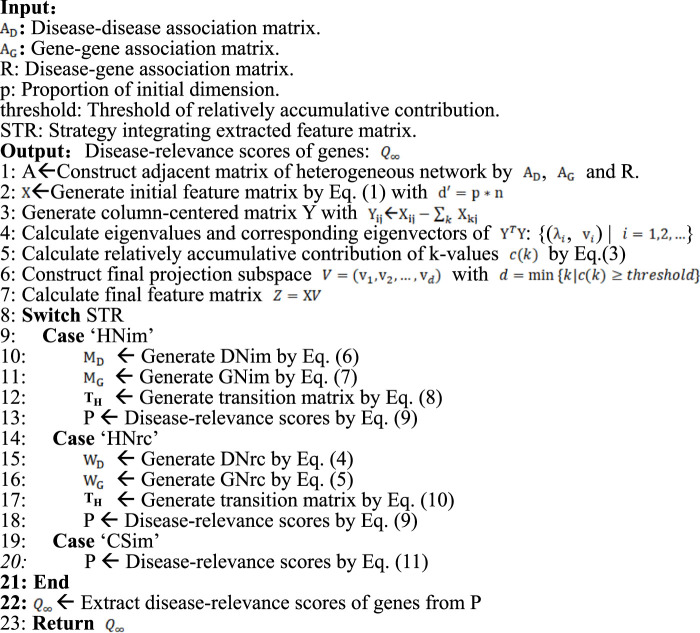


## 3 Results

### 3.1 Experimental setting and evaluation criteria

In this work, we evaluate the performance of our method by using the disease-gene network extracted from DisGeNet—one of the largest publicly available datasets of human pathogenic genes. We first evaluate the prediction performance of algorithms by 5-fold cross validation. Then, we evaluate the ability of our method in predicting the new added disease-gene associations by using the disease-gene associations before and after 2012 as the training set and the test set. Finally, we predict novel candidate genes for specific diseases by using all the known disease-gene associations as the training set, and we verify the disease relevance of the predicted candidates by literature verification and enrichment analysis.

For a disease d , we generate the disease-relevance scores of all candidate genes in our experiments. According to the decreasing order of the disease-relevance scores, we select the top-k genes as predicted positive genes for this disease, where k (e.g., k=1, 5 or 10 ) is a variable parameter. We use AUROC, AUPRC, Precision, Recall, F1-score, and Association Precision (AP) as evaluation criteria.

We used several state-of-the-art algorithms for disease-gene prediction as baseline methods, including PrGeFNE (Xiang et al., 2021b), dgn2vec (Liu et al., 2021), BiRW (Xie et al., 2012), RWRH (Li and Patra, 2010), PRINCE (Vanunu et al., 2010), DK (Köhler et al., 2008), RWR (Köhler et al., 2008). PrGeFNE and dgn2vec are a type of recently proposed network-based algorithms with network embedding, which use the network-embedding algorithms to extract features of nodes from a heterogeneous network and then predict pathogenic genes by using the extracted features of nodes.

### 3.2 Effect of different parameters in PGAGP

**FIGURE 2:**
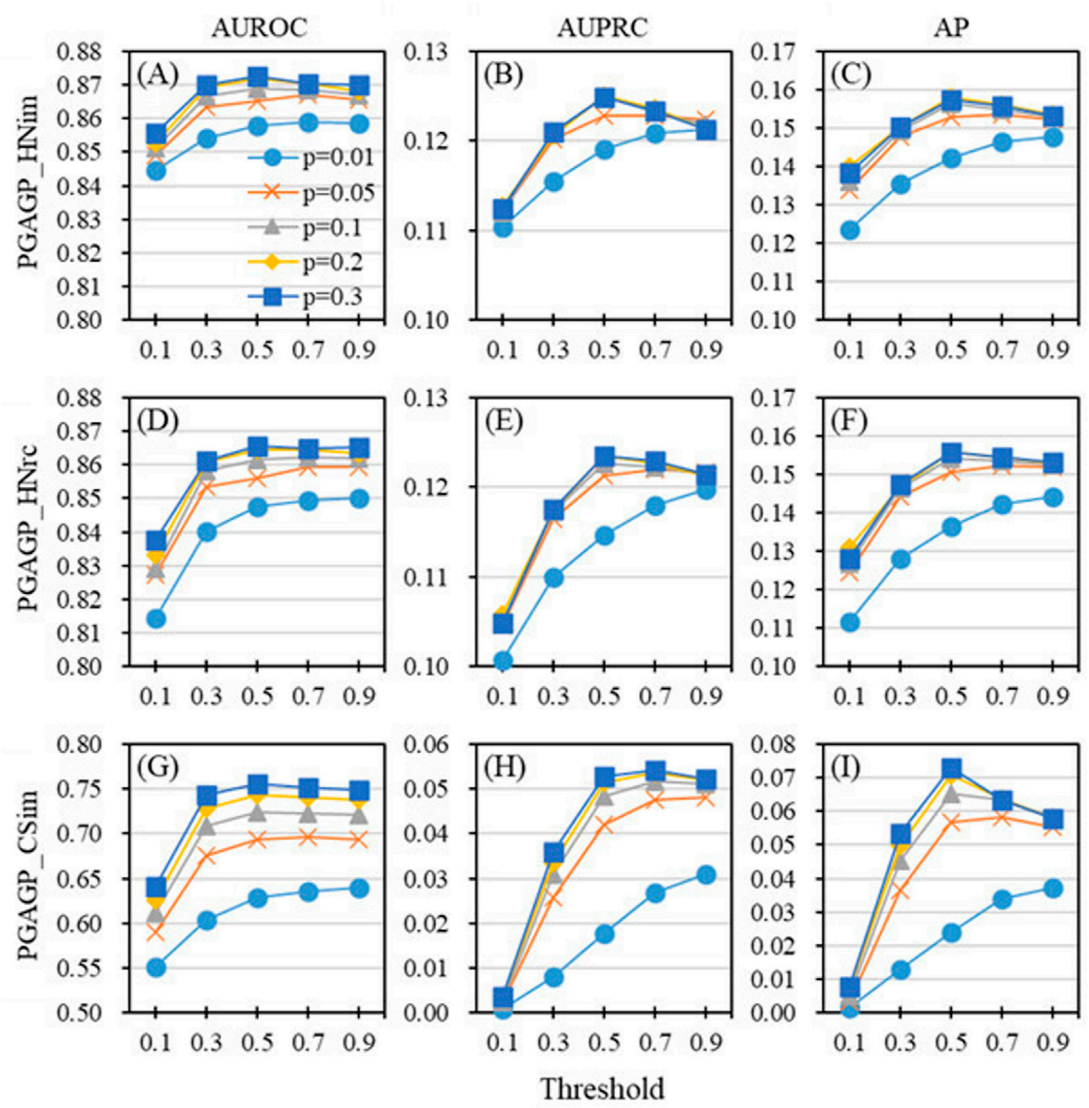
Effect of parameters on the performance of PGAGP **(A–C)** PGAGP_HNim **(D–F)** PGAGP_HNrc **(G–I)** PGAGP_CSim. For a given proportion of initial dimension reduction (*p*), different performance metrics (AUROC, AUPRC and AP) vary with the threshold of relatively accumulative contribution (threshold).

**FIGURE 3:**
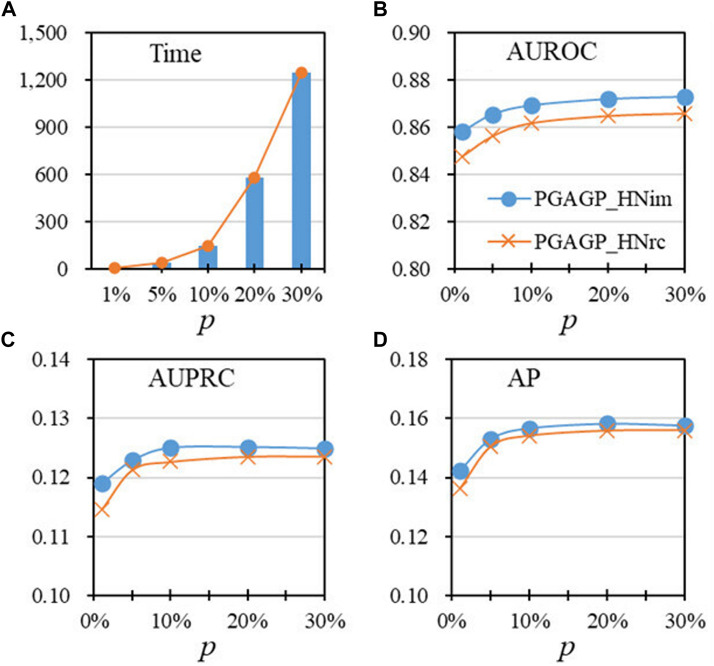
Performance as a function of the proportion of initial dimension (p), under the default threshold of relatively accumulative contribution **(A)** The running time for different values of *p* **(B**–**D)** AUROC, AUPRC and AP vary with the values of *p*.

We use the parameter *p* to determine the proportion of initial dimension and use the parameter ‘threshold’ to determine the retained relatively accumulative contribution. The two parameters play a crucial role in the adaptive refining process of generating the final low-dimensional feature matrix of nodes. Here, we study the effect of the two key parameters (*p* and threshold) on the performance of PGAGP (see Figures 2, 3).

Figure 2 shows the predictive performance of PGAGP’s three variants (PGAGP_HNim, PGAGP_HNrc and PGAGP_CSim) as a function of the threshold under given the values of *p*. For relatively small values of *p* (e.g., *p* = 0.01), the predictive performance metrics (AUROC, AUPRC and AP) will increase with the increase of the threshold. For relatively large values of *p* (e.g., *p* = 0.1, 0.2 and 0.3), the predictive performance metrics will first increase with the increase of the threshold, and then, after reaching a certain threshold (e.g., threshold = 0.5), they will be relatively stable, and even have a downward trend. So, based on the above relationship between prediction performance and threshold (especially when *p* is large), the threshold equal to 0.5 is worth recommending.

The possible reason for the above phenomenon is that when *p* is small (e.g., *p* = 0.01), the amount of information initially extracted from the original network by random projection itself is very small. Strong threshold filtering (smaller threshold) will lead to excessive loss of the information, leading to low prediction performance. With the increase of threshold, more and more information will be retained, which will gradually improve the prediction performance. In the case of small *p*, the influence of extracted (useful) information may be stronger than that of noise filtering.

However, when the parameter *p* is relatively large (e.g., *p* = 0.1, 0.2 and 0.3), the amount of information initially extracted from the original network by random projection is relatively abundant, which may also contain some useless noise information. Similarly, strong threshold filtering also corresponds to relatively low prediction performance, and when the threshold is increased, more and more information is retained, and the performance is gradually improved. Differently, when the threshold is raised to a certain extent (e.g., threshold = 0.5), more useless noise information is also retained, resulting in the decline of prediction performance. In other words, the weakening effect of retained useless information has been stronger than the positive effect of retained useful information. This also indicates that the refining process of threshold in AGP is useful for improve the ability of disease-gene prediction.

To study the effect of parameter *p*, Figure 3 shows the performance indicators of algorithms as a function of the proportion of initial dimension *p*), under the default threshold (=0.5). Figure 3A displays the running time of algorithms for different values of *p*, showing that the running time significantly increases with the increase of *p*, especially after *p* = 0.1; Figures 3B–D shows that PGAGP (PGAGP_HNim and PGAGP_HNrc) after *p* ≥ 0.1 has relatively stable and good performance (AUROC, AUPRC and AP), although AUROC and AP have still a trend of increase. Therefore, the proportion *p*) equal to 0.1 is recommended in this study due to its relatively low running time and good predictive performance.

### 3.3 Comparison of different strategies for integrating adaptive random projection

By considering three kind strategies (HNim, HNrc and CSim) for disease-gene prediction integrating adaptive random projection, we have proposed three variants of PGAGP (PGAGP_HNim, PGAGP_HNrc and PGAGP_CSim) to predict potential pathogenic genes. Table 1 and Figures 3B–D show the comparison of the three kind strategies corresponding to the three variants of PGAGP.

**TABLE 1:** Comparison with state-of-the-art algorithms in cross-validation experiments.

Methods	AUROC	AUPRC	Recall	Precision	F1-score	AP
PGAGP_HNim	**0.869**	**0.125**	**0.080**	**0.143**	**0.102**	**0.157**
PGAGP_HNrc	0.862	0.123	0.077	0.139	0.099	0.154
PGAGP_CSim	0.725	0.049	0.027	0.054	0.036	0.065
PrGeFNE	0.853	0.120	0.076	0.135	0.097	0.147
dgn2vec	0.829	0.064	0.036	0.051	0.042	0.051
RWRH	0.856	0.078	0.046	0.074	0.057	0.080
PRINCE	0.821	0.032	0.015	0.031	0.021	0.039
BiRW	0.768	0.046	0.027	0.045	0.034	0.046
DK	0.641	0.033	0.021	0.032	0.025	0.033
RWR	0.653	0.031	0.019	0.030	0.023	0.032

Bold values are the best among all the algorithms.

The results confirm that our proposed strategy (HNim) in this study has always been better than the other two existing strategies in literature (see Table 1 and Figures 3B–D). Therefore, HNim will be used as the recommended strategy integrating adaptive random projection, while PGAGP_HNim will be used as the recommended algorithm in this study.

### 3.4 Comparison to state-of-the-art algorithms

Here, our PGAGP method is compared with the state-of-the-art algorithms by cross-validation experiments: PrGeFNE (Xiang et al., 2021b), dgn2vec (Liu et al., 2021), RWRH (Li and Patra, 2010), PRINCE (Vanunu et al., 2010), BiRW (Xie et al., 2012), RWR (Köhler et al., 2008) and DK (Köhler et al., 2008). RWR uses a random walk process to explore the network proximity between candidate genes and seed genes (i.e., known pathogenic genes of a disease); DK is an algorithm based on a diffusion process on a PPI network; RWRH is the extension of RWR into disease-gene heterogeneous network; PRINCE is based on the network propagation process that makes use of the information of disease-disease associations; BiRW is based on the bi-random walk process on disease-gene heterogeneous network. Among these compared algorithms, RWR and DK are two popular algorithms based on PPI network; RWRH, PRINCE and BiRW are three widely used algorithms based on disease-gene heterogeneous network. PrGeFNE and dgn2vec are two recently proposed algorithms that integrate network embedding techniques. We used the preferred settings of our method (PGAGP_HNim with threshold = 0.5 and *p* = 0.1) in the following scenarios, while the default settings of the compared algorithms are used, which can be found in the original literature.

**FIGURE 4:**
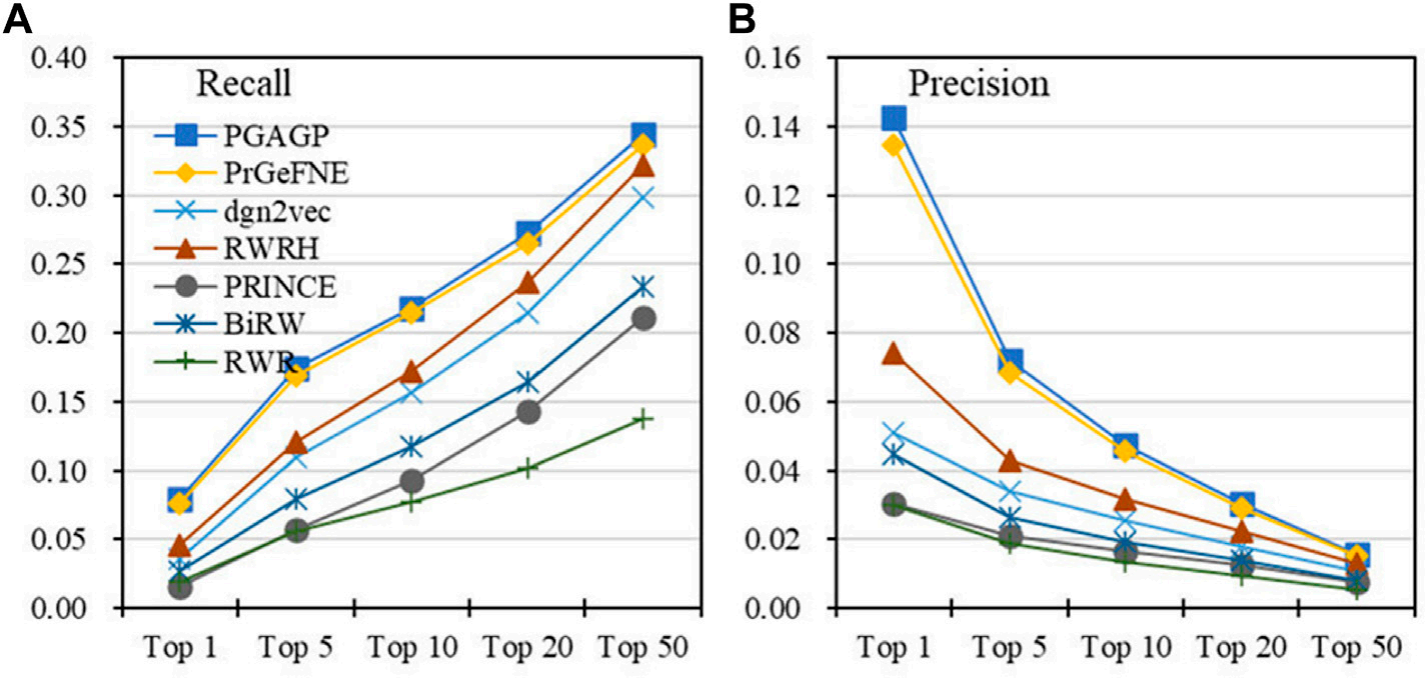
Top-k predictive performance of algorithms **(A)** Recall and **(B)** Precision in the top k of the prediction lists obtained by different algorithms.

Table 1 displays the AUROC, AUPRC, Recall, Precision, F1 and AP values of PGAGP (PGAGP_HNim, PGAGP_HNrc and PGAGP_CSim) and other comparison algorithms. Figure 4 displays the Recall and Precision in the top-k prediction lists obtained by different algorithms. These results show that our PGAGP algorithms (PGAGP_HNim and PGAGP_HNrc) outperform other comparison algorithms, including the recently proposed algorithms with network embedding techniques (PrGeFNE and dgn2vec).

PGAGP (PGAGP_HNim and PGAGP_HNrc) can be viewed as the improved versions of RWRH after combining network embedding. We can find that for AUROC, AUPRC, Recall, Precision, F1-score and AP, PGAGP_HNim is better than RWRH by 2%, 61%, 75%, 92%, 81% and 95%, respectively; PGAGP_HNrc is better than RWRH by 1%, 58%, 69%, 87%, 76% and 92%, respectively. Moreover, PGAGP_HNim is better than PrGeFNE by 2%, 4%, 5%, 6%, 5% and 6%, respectively, for AUROC, AUPRC, Recall, Precision, F1-score and AP; PGAGP_HNrc is better than PrGeFNE by 1%, 2%, 1%, 3%, 2% and 5%, respectively. Further, we can find that PGAGP_HNim exceeds the best results of comparison algorithms by 2%, 4%, 5%, 6%, 5% and 6%, respectively. Overall, these results indicate that our algorithms indeed can bring effective performance improvement.

### 3.5 Performance comparison in predicting new disease-gene associations

Further, we evaluate the performance of our PGAGP method algorithms (using default settings) by using the disease-gene associations before and after 2012 as a training set and a test set, respectively. Table 2 shows that our algorithm (PGAGP_HNim) also outperforms other state-of-the-art algorithms in predicting the newly added disease-gene associations.

**TABLE 2:** Comparison to state-of-the-art algorithms in predicting newly added disease-gene associations.

Methods	AUROC	AUPRC	Recall	Precision	F1-score	AP
PGAGP	**0.750**	**0.041**	**0.025**	**0.050**	**0.033**	**0.065**
PrGeFNE	0.737	0.039	0.023	0.043	0.030	0.064
dgn2vec	0.711	0.027	0.015	0.021	0.017	0.029
RWRH	0.747	0.025	0.014	0.024	0.017	0.036
PRINCE	0.718	0.012	0.005	0.010	0.007	0.018
CIPHER	0.569	0.009	0.005	0.009	0.006	0.005
BiRW	0.690	0.016	0.009	0.014	0.011	0.016
RWR	0.585	0.012	0.008	0.013	0.010	0.016
DK	0.577	0.009	0.005	0.006	0.006	0.015

Bold values are the best among all the algorithms.

For example, specifically, we can find that PGAGP_HNim is better than RWRH by 0.3%, 62%, 84%, 110%, 93% and 80%, respectively, for AUROC, AUPRC, Recall, Precision, F1 and AP; PGAGP_HNim is better than PrGeFNE by 2%, 6%, 9%, 17%, 12% and 1%, respectively; PGAGP_HNim exceeds the best results of comparison algorithms by 0.3%, 6%, 9%, 17%, 12% and 1%, respectively. The results indicate that our PGAGP algorithm can also bring performance improvement in predicting newly added disease-gene associations, again verifying the effectiveness of PGAGP.

### 3.6 Comparison with other network embedding algorithms

Here, we further evaluate the effectiveness of our AGP network-embedding algorithm in our PGAGP framework, by comparing it with other state-of-art network embedding algorithms (Han et al., 2018): dgn2vec (Liu et al., 2021), RandNE (Zhang et al., 2018), node2vec (Grover and Leskovec, 2016), SDNE (Wang et al., 2016), LINE (Tang et al., 2015), GraRep (Cao et al., 2015), DeepWalk (Perozzi et al., 2014), Graph Factorization (GF) (Ahmed et al., 2013), Laplacian Eigenmaps (LAP) (Belkin and Niyogi, 2001), and LLE (Roweis and Saul, 2000). We also used the preferred settings of our method in the following scenarios, while the default settings of the compared network-embedding algorithms are used, which can be found, e.g., in the OpenNE package or the original literature.

The following is a brief introduction to the state-of-art network embedding algorithms. dgn2vec is a network-embedding algorithm on disease-gene heterogeneous network, which was presented for disease-gene prediction in another literature. RandNE is a network-embedding algorithm based on iterative random projection, which was proposed for billion-scale network embedding. DeepWalk is the first learning-based algorithm, which learns the vector representation of network nodes by the Skip-gram word embedding model. In this algorithm, network nodes are compared to word in the language model, and the node sequence generated by random walks is regarded as the context. By predicting the random walk sequence of selected nodes, the parameters of the probability model are estimated to obtain the node embedding representation. LINE considers the first order and second order proximity of network nodes at the same time, which is mainly manifested as the high proximity of two directly connected nodes and the high proximity of two nodes with more common neighbors. node2vec is the improved algorithm based on DeepWalk. node2vec combines depth-first search (DFS) and breadth-first search (BFS) to conduct “biased” random walks, generate node sequence sets, and then use them as the input of the Skip-gram to get network embedding. GraRep conducts matrix decomposition on the adjacency matrix of the network and its higher-order power, so as to obtain the representation of the network nodes by using different levels of neighbor node information. SDNE combines the semi-supervised deep learning model of the first order and second order proximity of the optimized network, while preserving the global and local structure information of the network. GF is a graph-factorization algorithm for large-scale graph decomposition and inference. LAP is a geometrically motivated algorithm for representing the high-dimensional data, which is a computationally efficient algorithm to non-linear dimensionality reduction. LLE is a locally linear embedding algorithm based on unsupervised learning for non-linear dimensionality reduction, which can compute low-dimensional, neighborhood-preserving embeddings of high-dimensional data.

In the framework of PGAGP, we compared the predictive performance of the AGP algorithm with these state-of-the-art network-embedding algorithms. Experimental results show that the PGAGP framework with different network-embedding algorithms can improve the ability of predicting disease genes, especially for AUPRC, Recall, Precision, F1-score and AP, compared to the baseline algorithm; and AGP can obtain relatively better results than other network-embedding algorithms in many cases (see Table 3).

**TABLE 3:** Comparison of different network embedding algorithms (AGP and other state-of-the-art algorithms) in the framework of PGAGP (using HNim strategy).

Methods	AUROC	AUPRC	Recall	Precision	F1-score	AP
AGP	0.869	**0.125**	**0.080**	**0.143**	**0.102**	**0.157**
dgn2vec	0.873	0.123	0.077	0.135	0.098	0.148
RandNE	0.862	0.122	0.078	0.137	0.099	0.150
LINE	0.836	0.109	0.072	0.117	0.089	0.122
node2vec	0.870	0.124	0.079	0.139	0.100	0.153
SDNE	0.841	0.110	0.072	0.119	0.090	0.127
DeepWalk	**0.877**	0.121	0.076	0.135	0.097	0.151
GraRep	0.863	0.114	0.072	0.127	0.092	0.144
GF	0.851	0.121	0.079	0.131	0.098	0.140
LAP	0.856	0.114	0.072	0.125	0.091	0.142
LLE	0.856	0.108	0.069	0.116	0.086	0.130
Baseline	0.856	0.078	0.046	0.074	0.057	0.080

Bold values are the best among all the algorithms.

As we know, the AGP algorithm is proposed based on Gaussian random projection (GRP). Compared to another GRP-based algorithm called RandNE, we can see that the performance of AGP is improved by 1%, 2%, 2%, 4%, 3% and 4% for AUROC, AUPRC, Recall, Precision, F1-score and AP. The reason of the improvement may be that RandNE directly applies the (iterative) GRP to obtain the features of nodes from a large-size network, while AGP consists of two key steps: the initial feature extraction by GRP and the optimization of the features by an adaptive refining process.

## 4 Case study

Here, we use all known disease-gene associations as train set and perform our PGAGP algorithm to score all candidate genes for specific diseases including Alzheimer’s disease (AD) and Parkinson’s disease (PD). Then, the ranking lists of candidate genes were generated by the decreasing genes’ scores. The higher the gene rank, the more likely it is to be associated with disease. The top-10 predicted candidate genes were listed in Tables 4, 5. According to the prediction algorithm scores, these genes are expected to be the most closely related to the diseases among all candidate genes. To check the disease relatedness of these candidate genes, we tried to find associations between candidate genes and related diseases by searching the literature.

**TABLE 4:** Predicted top 10 candidate genes for Alzheimer’s Disease.

Rank	Candidate gene	References
1	*GRN*	Viswanathan et al. (2009); Kämäläinen et al. (2013)
2	*IL6*	Licastro et al. (2000); Chen et al. (2012); Qi et al. (2012)
3	*IFNG*	—
4	*POMC*	Shen et al. (2016); Zamanian-Azodi et al. (2020)
5	*PAH*	—
6	*EDN1*	Palmer et al. (2012); Thomas et al. (2015); Alcendor. (2020)
7	*NOS2*	Wilcock et al. (2008); Colton et al. (2014)
8	*CAT*	—
9	*SOD1*	Feng et al. (2006); Spisak et al. (2014)
10	*ALB*	—

**TABLE 5:** Predicted top 10 candidate genes for Parkinson’s disease.

Rank	Candidate gene	References
1	*APOE*	de la Fuente-Fernández et al. (1999); Li et al. (2018)
2	*PDYN*	—
3	*PODXL*	—
4	*NOS3*	—
5	*IL1B*	Mattila et al. (2002); Nishimura et al. (2005); Lee et al. (2016)
6	*CAT*	—
7	*NOS2*	Hancock et al. (2008)
8	*DNAJC13*	Vilariño-Güell et al. (2014); Gustavsson et al. (2015)
9	*GSR*	—
10	*APP*	Schulte et al. (2015); Zeng et al. (2022)

### 4.1 Alzheimer’s disease

Alzheimer’s disease (AD) is a neurodegenerative disease common in the elderly (especially over 65 years old). Its pathological features are progressive hippocampal neuron loss and memory dysfunction. At present, there are many hypotheses about the pathogenesis of AD, including β-amyloid (Aβ) Tau protein hyperphosphorylation, excitatory amino acids, genes, chronic inflammation, neurodegeneration caused by oxygen free radicals, brain neuron apoptosis (Hardy and Selkoe, 2002). The top-10 predicted genes were listed in Table 4. To check the disease relatedness of these candidate genes, we tried to find associations between candidate genes and AD by searching the literature.

Inflammatory pathological changes of AD, glial cell-mediated inflammation and overexpression of inflammatory cytokines in the brain have shown that inflammatory reaction plays an important role in the formation and development of AD (Bolós et al., 2017). Aβ protein in the brains of AD patients acts as an inflammatory stimulator, activating astrocyte and microglia to release inflammatory cytokines including IL-1β, IL-6 and TNF-α, which may be one of the main pathogeneses of AD (Ng et al., 2018). Licastro et al., have reported that polymorphism of the IL-6 gene was a risk factor for late-onset AD (Licastro et al., 2000). While Chen et al., indicated that the variants of IL-6 gene were protective factors for late-onset AD (Chen et al., 2012). In addition, a meta-analysis has revealed that two polymorphisms in IL-6 gene including -174 G/C and -572 C/G were risk factor for AD. Furthermore, the nitric oxide synthase 2 (NOS2), that encoding the inducible NOS (iNOS) has been reported to play an important role in neuroinflammation (Colton et al., 2008). Researchers have shown that removal of NOS2 gene from an APP transgenic mouse results in development of a much greater spectrum of AD-like pathology and behavioral impairments (Wilcock et al., 2008; Colton et al., 2014).

In addition, oxidative stress is a peroxidative state caused by imbalance of oxidative and antioxidant components in the body, which can accelerate human aging and is related to many pathological processes such as AD (Zhu et al., 2004). Overexpression of malondialdehyde (MDA) and superoxide dismutase (SOD) suggested that oxidative stress plays an important role in the formation and development of AD (Spisak et al., 2014).

As for Granulins (GRN) gene, the GRN rs5848A could reduce plasma granulin levels in AD cohort (Kämäläinen et al., 2013). In addition, genetic variability in the GRN gene variants was also reported to be associated with the risk of AD in a Finnish population (Viswanathan et al., 2009). In addition, pro-opiomelanocortin (POMC)-derived neuropeptides and melanocortin four receptor (MC4R) were shown to implicate in hippocampus-dependent synaptic plasticity. Disruption of the hippocampal POMC/MC4R circuit might contribute to synaptic dysfunction observed in AD (Shen et al., 2016). The POMC gene expression was significantly different in the treated AD mice with ibuprofen relative to the AD mice (Zamanian-Azodi et al., 2020). Furthermore, the endothelin system plays potential role in AD. ET-1 was one of the most important member of ETs proteins (Alcendor, 2020). ET-1 was encoded by endothelin-1 (EDN1) gene, which was demonstrated to elevated in AD and upregulated by Amyloid-β (Palmer et al., 2012). In addition, ET-1 has been shown to result in neuronal injury in AD (Thomas et al., 2015). The above results may imply that the predictions were similar to those of existing studies. And the algorithm was valuable for predicting the new disease-gene associations.

**FIGURE 5:**
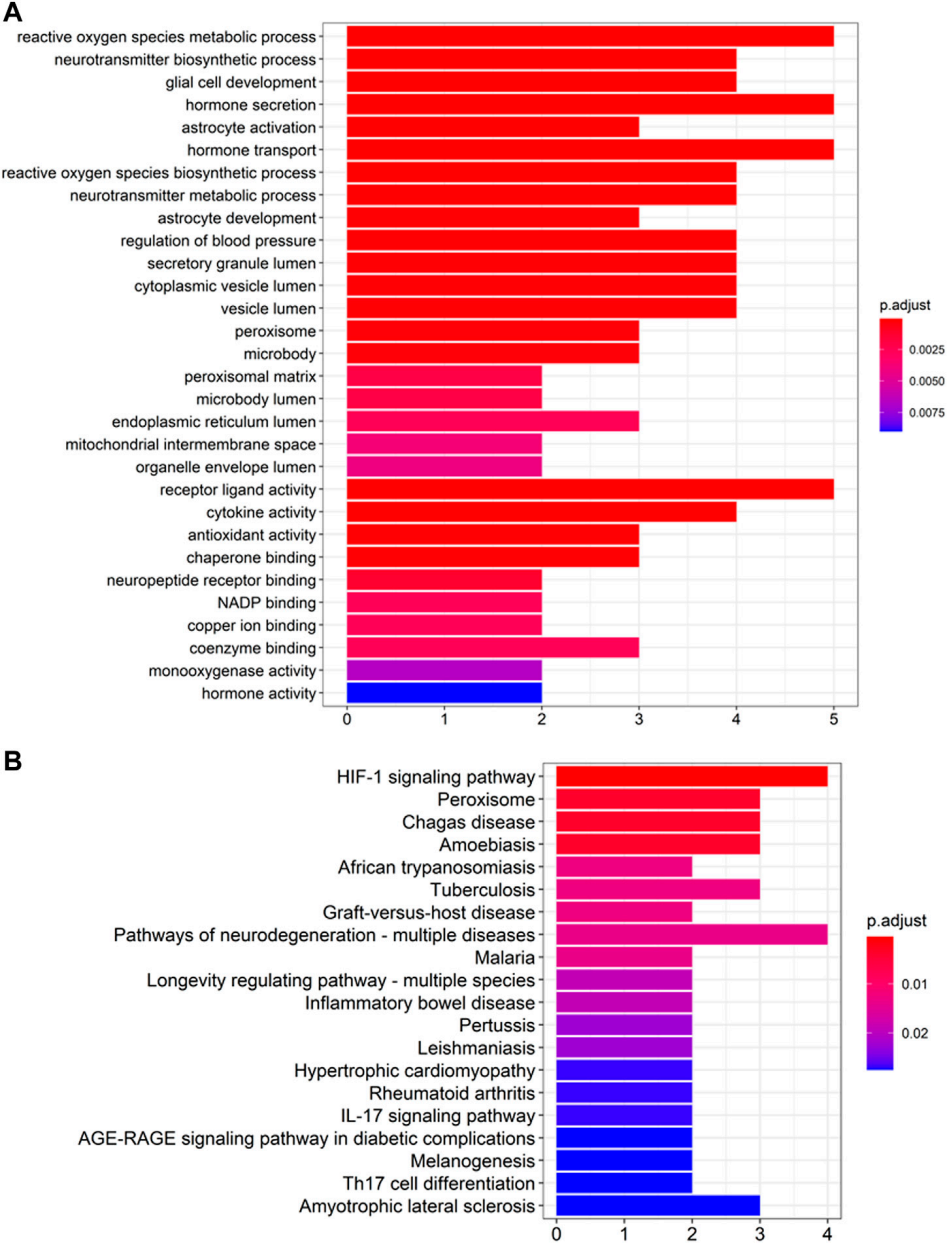
Enrichment analysis of top 10 candidate genes for Alzheimer’s Disease **(A)** GO enrichment analysis and **(B)** KEGG pathway enrichment analysis.

The GO and KEGG pathway enrichment analysis on the top 10 ranked genes were performed to evaluate the predictions. GO enrichment showed that the genes were enriched in the BP of glial cell development and neurotransmitter biosynthetic process, and in the CC of peroxisome, vesicle lumen, and secretory granule lumen, as well as in the MF of chaperone binding, antioxidant activity, and cytokine activity (see Figure 5A). Additionally, KEGG pathway enrichment implied that the genes were mostly enriched in amyotrophic lateral sclerosis, pathways of neurodegeneration-multiple diseases and HIF-1 pathways, which were shown to be associated with the pathology of AD (see Figure 5B).

### 4.2 Parkinson’s disease

Parkinson’s disease (PD) is a common degenerative disease of the central nervous system (CNS), which is mainly characterized by the degeneration and loss of dopamine neurons in the substantia nigra and striatum of the brainstem.

The all known disease genes were used as train set to predict candidate genes, by using improved algorithm PGAGP. The top-10 predicted genes were listed in Table 5.

*ApoE* gene was located at 19q13.32. Studies have shown that ApoE rs429358 and rs7412 were associated with PD. Fuente-fernndez et al. (de la Fuente-Fernández et al., 1999)found that *ApoE* gene polymorphism was associated with hallucinatory symptoms in PD patients without dementia. In addition, a meta-analysis of 47 studies found that the *ApoE* allele may be a risk factor for hallucination susceptibility in Asian PD population (Li et al., 2018).

IL1 has been illustrated to have a role in PD. Variation in the *IL1α, IL1β,* and *IL1RN* genes may be of importance in the development of this disorder. Evidence has shown that the *IL1β* (−511) *1/*1 genotype was a risk factor on age at onset of PD (Mattila et al., 2002; Nishimura et al., 2005). Lee et al. have reported genetic variation (rs16944) in the proinflammatory cytokine gene *IL1β* contribute to risk of developing PD (Lee et al., 2016).

As for *NOS2* gene, studies have reported that the variants in *NOS2A*gene were associated with PD risk (Hancock et al., 2006). In addition, multiple polymorphisms in NOS2A gene including rs2072324, rs944725, rs12944039, rs2248814, rs2297516, rs1060826, and rs2255929, were significantly associated with PD, particularly in earlier-onset families with sporadic PD (Hancock et al., 2008).

The protein encoded by heat shock protein 40 homologous subtype 13 (DNAJC13) is involved in the transport of early endosomes, the cycle of endocytic vesicles, and the lysosomal enzymatic hydrolysis pathway. It is currently believed that molecular defects in these processes are directly related to the pathogenesis of PD. Vilario-guell et al. (Vilariño-Güell et al., 2014)conducted gene sequencing on 2928 PD patients from Canada, Norway, Taiwan, Tunisia and the United States, and found that the mutation *p*. ASN855ser was closely related to its pathogenesis. Recently, Gustavsson et al. (Gustavsson et al., 2015) conducted gene sequencing on 201 PD patients and found that the following variants existed: P. E1740Q, p. R1516h, p. N855S, p. L2170W, p. P336a, p. V722L, p. R1266q, in addition to P.N855S, other rare variants may increase the susceptibility of the disease.

β-amyloid precursor protein (Rivas et al., 2015) is the precursor of Aβ. Recently, some variants of AD-causal genes including *APP* have been reported in PD (Mota et al., 2019; Zeng et al., 2022). Schulte et al. have shown that rare variants in *APP* gene were more common in PD cases overall than in either the AD cases or controls. And a rare variant in *APP* gene (c.1795G>A (p (E599K))) was revealed to be significantly associated with the PD phenotype (Schulte et al., 2015).

**FIGURE 6:**
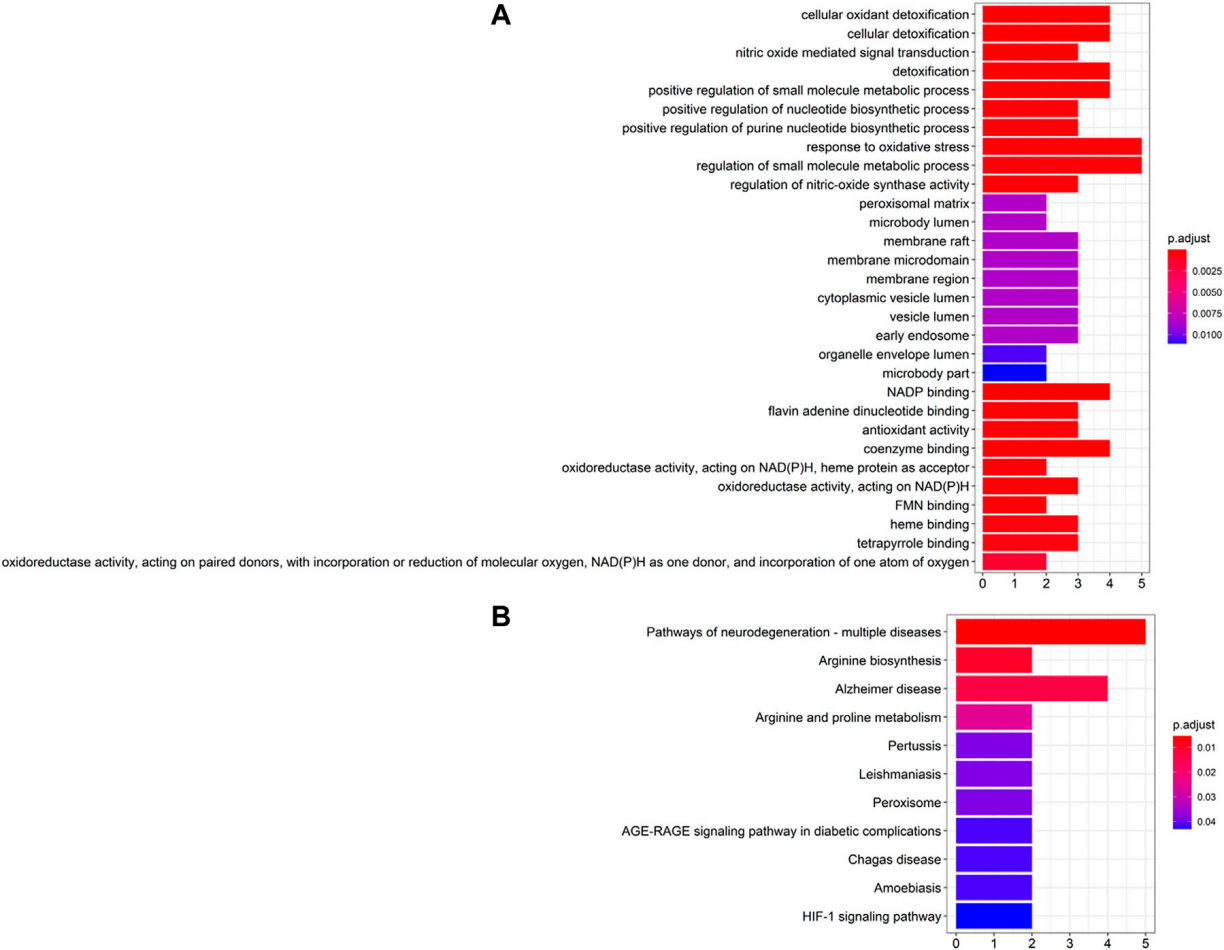
Enrichment analysis of top 10 candidate genes for Parkinson’s disease. **(A)** GO enrichment analysis and **(B)** KEGG pathway enrichment analysis.

The GO and KEGG pathway enrichment analysis on the top 10 ranked genes were performed. GO enrichment analysis showed that genes were enriched in the BP of response to oxidative stress, neurotransmitter biosynthetic process, and amyloid fibril formation, and in the CC of peroxisome, astrocyte projection, and neuronal cell body, as well as in the MF of oxidoreductase activity, antioxidant activity, and tau protein binding (see Figure 6A). Additionally, KEGG pathway enrichment suggested that the genes were mostly enriched in pathways of neurodegeneration, Alzheimer disease, arginine biosynthesis and peroxisome pathways (see Figure 6B).

## 5 Conclusion

The emergence and development of diseases are a complex process related to the mutation and dysfunction of genes. It is of great significance to study the molecular mechanism of diseases by integrating the association data of multiple types of biological entities. In this paper, we have proposed a type of novel methods called PGAGP for disease-gene prediction by the AGP algorithm that combines Gaussian random projection and an adaptive refining process, which can make use of disease-gene heterogeneous network to effectively enhance the ability of disease-gene prediction.

We have systematically studied the effect of PGAGP’s parameters and different strategies (HNim, HNrc and CSim) of integrating adaptive random projection on the predictive performance, by which PGAGP with effective parameters and strategy (PGAGP_HNim) is determined. Specifically, PGAGP_HNim first constructs a disease-gene heterogeneous network by using PPIs, disease-disease associations and disease-gene associations; then, it uses the AGP network-embedding algorithm to more effectively extract the low-dimensional features of nodes from the network; finally, an improved disease-gene heterogenous network is constructed by using the low-dimensional features, and the random walk with restart is applied to the improved heterogenous network so as to predict disease genes more effectively.

We have confirmed that PGAGP outperforms the state-of-the-art algorithms by the cross-validation experiments as well as test of newly added associations. We also have compared the AGP network embedding algorithm with other state-of-the-art network embedding algorithms under the framework of PGAGP_HNim and show that AGP outperforms these compared network-embedding algorithms in many cases. Finally, the case studies for specific diseases such as Alzheimer Disease and Parkinson Disease have been conducted, which further confirm the effectiveness of our method since many of the predicted candidate genes for these diseases have been implied to be related to these diseases by literature verification and enrichment analysis.

Overall, we have provided an effective solution for integrating AGP network embedding to predict disease genes more effectively. This work can inspire the solution of related tasks in bioinformatics such as miRNA-disease association prediction or lncRNA-disease association prediction.

## Data Availability

The original contributions presented in the study are included in the article/supplementary material, further inquiries can be directed to the corresponding authors.
